# Modifier locus mapping of a transgenic F2 mouse population identifies *CCDC115* as a novel aggressive prostate cancer modifier gene in humans

**DOI:** 10.1186/s12864-018-4827-2

**Published:** 2018-06-11

**Authors:** Jean M. Winter, Natasha L. Curry, Derek M. Gildea, Kendra A. Williams, Minnkyong Lee, Ying Hu, Nigel P. S. Crawford

**Affiliations:** 1 Metastasis Genetics Section, Genetics and Molecular Biology Branch, National Human Genome Research Institute, NIH, Bethesda, MD 20892 USA; 2 Computational and Statistical Genomics Branch, National Human Genome Research Institute, NIH, Bethesda, MD 20892 USA; 3 Center for Biomedical Informatics and Information Technology, National Cancer Institute, NIH, Rockville, MD 20892 USA; 4 Present address: Dame Roma Mitchell Cancer Research Laboratories, Adelaide Health and Medical Sciences, The University of Adelaide, Adelaide, South Australia 5000 Australia; 5 Present address: Sanofi, 55 Corporate Dr., Bridgewater, NJ 08897 USA

**Keywords:** Prostate cancer, Germline variation, Quantitative trait loci, *CCDC115*, *DNAJC10*, *RNF149*, *STYXL1*, LNCaP, TRAMP mouse model

## Abstract

**Background:**

It is well known that development of prostate cancer (PC) can be attributed to somatic mutations of the genome, acquired within proto-oncogenes or tumor-suppressor genes. What is less well understood is how germline variation contributes to disease aggressiveness in PC patients. To map germline modifiers of aggressive neuroendocrine PC, we generated a genetically diverse F2 intercross population using the transgenic TRAMP mouse model and the wild-derived WSB/EiJ (WSB) strain. The relevance of germline modifiers of aggressive PC identified in these mice was extensively correlated in human PC datasets and functionally validated in cell lines.

**Results:**

Aggressive PC traits were quantified in a population of 30 week old (TRAMP x WSB) F2 mice (*n* = 307). Correlation of germline genotype with aggressive disease phenotype revealed seven modifier loci that were significantly associated with aggressive disease. RNA-seq were analyzed using cis-eQTL and trait correlation analyses to identify candidate genes within each of these loci. Analysis of 92 (TRAMP x WSB) F2 prostates revealed 25 candidate genes that harbored both a significant *cis*-eQTL and mRNA expression correlations with an aggressive PC trait. We further delineated these candidate genes based on their clinical relevance, by interrogating human PC GWAS and PC tumor gene expression datasets. We identified four genes (*CCDC115, DNAJC10, RNF149, and STYXL1*), which encompassed all of the following characteristics: 1) one or more germline variants associated with aggressive PC traits; 2) differential mRNA levels associated with aggressive PC traits; and 3) differential mRNA expression between normal and tumor tissue. Functional validation studies of these four genes using the human LNCaP prostate adenocarcinoma cell line revealed ectopic overexpression of *CCDC115* can significantly impede cell growth in vitro and tumor growth in vivo*.* Furthermore, *CCDC115* human prostate tumor expression was associated with better survival outcomes.

**Conclusion:**

We have demonstrated how modifier locus mapping in mouse models of PC, coupled with in silico analyses of human PC datasets, can reveal novel germline modifier genes of aggressive PC. We have also characterized *CCDC115* as being associated with less aggressive PC in humans, placing it as a potential prognostic marker of aggressive PC.

**Electronic supplementary material:**

The online version of this article (10.1186/s12864-018-4827-2) contains supplementary material, which is available to authorized users.

## Background

Prostate cancer (PC) is the most commonly diagnosed cancer in the USA, with an estimated 161,360 men expected to be diagnosed in 2017, and 26,730 men are anticipated to die from PC in 2017 [1]. The means of assessing prognosis at the time of diagnosis are inaccurate. For example, measuring elevated serum levels of the prostate specific antigen (PSA), a test that has been used in routine PC screening for several decades, cannot stratify patients into low and high risk categories, and therefore cannot determine which individuals are likely to have disease progression or those that have indolent versus aggressive pathology at the time of diagnosis. These inaccuracies increase the likelihood that men with low-grade disease will undergo treatments associated with high rates of morbidity [2]. This indicates that screening increases detection of indolent, low grade tumors, and patients unnecessarily suffer through treatments for tumors that would otherwise go undiagnosed with no apparent effect on survival. Therefore, there is a need to develop new clinical tools to distinguish those patients at low or high risk at the time of diagnosis, in order to better direct treatment options.

Genetics plays an integral role in determining individual risk for developing PC. It’s well known that PC develops as a result of somatic mutations of proto-oncogenes and/or tumor-suppressor genes, which drive tumorigenesis of prostate epithelial cells into adenocarcinoma over the course of many years. However, some prostate adenocarcinomas can evade aggressive therapeutic treatment strategies, where they disseminate to distant sites forming a castrate-resistant disease state termed neuroendocrine prostate cancer (NEPC) [3, 4]. Although NEPC only accounts for approximately 1% of new PC diagnoses, it is highly aggressive, does not respond to current treatment regimens and is usually fatal. Recent innovations in genomic technologies have led to identification of somatic and germline risk loci associated with PC susceptibility. Somatic alterations of the *RB1*, *TP53*, *and PTEN* genes have been identified as determinants of NEPC development, as well as overexpression and amplification of both *MYCN* and *AURKA* [5–7]. However, it is less clearly understood how germline variation can influence late stage disease processes and ultimately impact on an individual’s risk of developing the more aggressive, fatal form of NEPC. Family-based linkage studies have proven somewhat challenging due to the heterogeneous nature of PC, but have denoted the presence of multiple hereditary genetic loci associated with aggressive disease susceptibility [8]. Additionally, genome-wide association studies (GWAS) of PC have revealed over one hundred variants associated with PC development risk loci [9]. However, GWAS have revealed only a few variants associated with PC aggressiveness [10, 11]. This is likely a reflection of the difficulties of assessing biological effects at late disease stages. Cofounding factors such as different environmental exposures, smaller sample sizes, case-control overlap and strict parameters for multiple testing means there is large proportion of “missing heritability” in GWAS [12]. Thus, many of the variants identified using GWAS that do not reach the stringent genome wide significance may likely still possess biologically meaningful association with clinical outcome [12]. New approaches that augment GAWS data are needed to uncover consequential variants of aggressive PC.

To overcome these hurdles, systems genetics approaches have been successfully implemented to identify human aggressive PC modifier genes [13] .Our lab first demonstrated that hereditary variants can influence aggressive disease in a transgenic mouse model of NEPC, the C57BL/6-Tg(TRAMP)8247Ng/J (TRAMP) mouse [14]. In a ‘proof of principle’ experiment, we crossed 8 strains of inbred mice with the TRAMP mouse, and demonstrated that phenotypic traits of aggressive PC varied considerably depending on genetic background [14]. This seminal work revealed a strong correlation between tumor growth (weight) in the TRAMP mouse with lower age of euthanasia and higher incidence of metastases, both locally to regional lymph nodes and distant metastasis to visceral organs. Given these findings, we bred F2 mouse populations of two of the 8 strains ((TRAMP x PWK/PhJ) F2 and (TRAMP x NOD/ShiLtJ) F2). Quantitative trait loci (QTL) mapping coupled with systems genetics approaches in mice and human populations revealed several novel modifiers of aggressive PC [15, 16]. In a more complex study, we used a similar genetics approach using an F1 population bred from the TRAMP mouse and Diversity Outbred mice [17], with the latter being a highly genetically diverse mouse strain harboring over 40 million single nucleotide polymorphisms (SNPs), derived from the same 8 inbred strains used in our strain survey. High resolution fine mapping of QTLs in these mice, coupled with analysis of human PC GWAS and PC gene expression datasets, revealed novel germline modifiers of aggressive PC [18]. These data demonstrate that systems genetics approaches centering on genetically diverse transgenic mouse models of NEPC can reveal novel modifiers of aggressive PC that would otherwise only reach nominal but not genome wide significance in GWAS studies.

Previously, in our 8 mouse strain survey [14], we identified that (TRAMP x WSB/EiJ) F1 mice had significantly reduced primary tumor burden but had an increased metastatic burden compared to the wildtype C57BL/6 J TRAMP mouse. The study we present here utilizes an F2 population bred from C57BL/6 J TRAMP and WSB/EiJ mice to delineate genetic variants that modulate aggressive NEPC. The TRAMP mouse model, although not encompassing all neuroendocrine differentiation observed in humans, does mimic small cell NEPC, and utilizes the SV40 T-antigen oncoprotein expression driven by the androgen-responsive minimal probasin promoter (PB). It is ideal for mapping aggressive disease traits of NEPC since their primary tumors harbor many biological similarities to human disease [5, 19, 20], including metastasis to visceral organs [21, 22]. We aimed to uncover novel variants associated with aggressive traits of PC in a (TRAMP x WSB/EiJ) F2 mouse population using a systems genetics approach. We aimed to validate these candidate genes by characterizing the biological impact of their overexpression on tumor growth and metastasis. Overall, this study provides further insights into the mechanistic role of germline variants on aggressive and fatal forms PC.

## Methods

### Mouse experimental strategy and tissue sampling

**Fig. 1:**
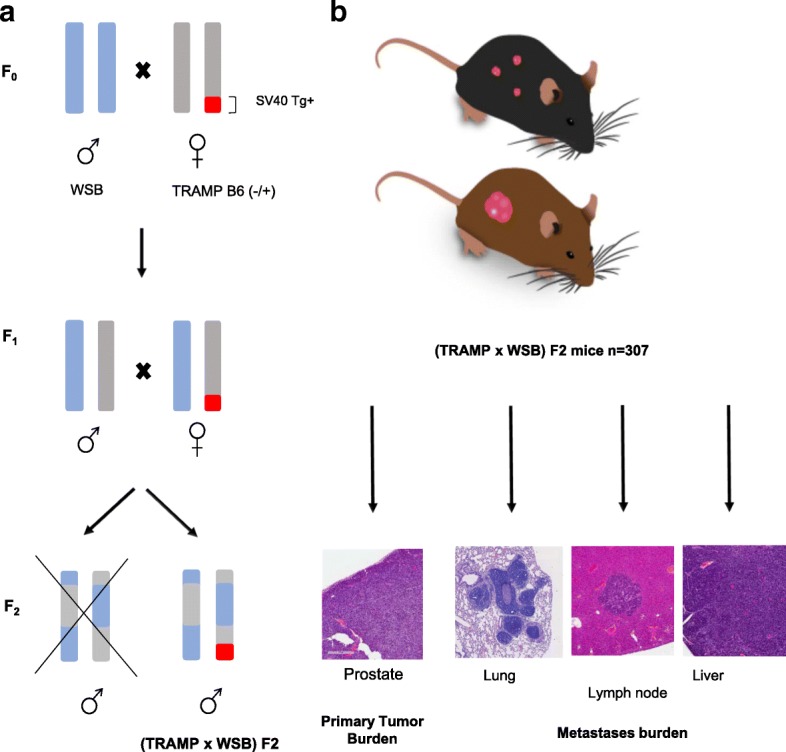
Schematic outline of mouse experimental study. **a** Mouse breeding strategy to produce experimental (TRAMP x WSB) F2 mouse population. WSB male and female mice were bred to generate an F1 population of WSB mice. Male WSB F1 mice were then crossed with SV40 transgene positive TRAMP B6 female mice to generate an F2 population of WSB mice that are SV40 Transgene positive (Tg+). **b** A total of 307 male (TRAMP x WSB) F2 mice were maintained for 210 days, or until humane endpoints were reached, and sacrificed to quantify phenotypic traits of aggressive PC, including primary tumor and metastasis burden. These phenotypic traits were used further in genomic and transcriptomic analyses to identify loci associated with aggressive disease traits in (TRAMP x WXB) F2 mice

An outline of our approach to the F2 mouse breeding scheme is presented in Fig. 1a. Male WSB/EiJ (WSB) mice (The Jackson Laboratories, Bar Harbor, ME) were crossed with female C57BL/6 J-Tg(TRAMP)824Ng/J (TRAMP) mice (The Jackson Laboratories, Bar Harbor, ME) to generate (TRAMP x WSB) F1 offspring. Male and female (TRAMP x WSB) F1 mice were weaned at 21 days and tail tissue collected for genotyping of the SV40 T Antigen (Tg) oncogene using the HotSHOT method [23]. F2 mice were generated by crossing Tg-positive F1 females with Tg-negative F1 males. Male Tg-positive (TRAMP x WSB) F2 progeny were subsequently used for downstream phenotypic trait quantification, as outlined in Fig. 1b. A total of *n* = 307 male (TRAMP x WSB) F2 mice were fed ad libitum*,* housed with a maximum of 5 per cage under controlled conditions of 22 ± 2 °C, 80 ± 10% humidity and 12-h light/dark cycle and monitored daily over 210 days for signs of distress with human end points classified as palpable tumor larger than 20 mm, rapid weight loss, hunched posture, labored breathing, trauma, impaired mobility, dysuria, or difficulty in obtaining food or water.

At time of euthanasia mice were sacrificed by pentobarbital injection and prostates were carefully resected, being cautious not to rupture the seminal vesicles. The weight of prostate and seminal vesicles were recorded to quantify tumor burden (in grams). The prostate was sectioned into smaller pieces and snap frozen in liquid nitrogen and stored at − 80 °C prior to RNA extraction. Macroscopic metastatic tumors of the lung and liver were counted, and enlarged para-aortic lymph nodes were counted and weighed, with whole tissues collected in 10% *w*/*v* phosphate buffered formaldehyde and processed for hematoxylin and eosin staining. Histology slides were scanned with Scanscope Digital microscope (Aperio, Vista, CA). For high density SNP genotyping, tail biopsies were collected for DNA extraction. Mean and standard deviation (SD) of prostate and seminal vesicle burden (grams) were calculated for all 307 mice, while metastases were calculated as incidence (%) of mice harboring metastases. Prostate and seminal vesicle weight (ie tumour burden) were used in downstream QTL mapping analyses, without confirming pathological evidence of tumor, since we have shown previously that organ weight is highly correlated with presence of cancer [14]. All animals were handled, housed and used in the experiments humanely in accordance with the NHGRI Animal Care and Use Committee guidelines under animal study protocol G-09-2.

### QTL mapping using high density SNP genotyping

Methods of SNP genotyping described here have been performed in previously published work [15, 16]. Purified genomic DNA sampled from mouse tail biopsies was obtained using standard phenol chloroform extraction and concentration was assessed using the Nanodrop 2000 (Thermosfisher). A total of 5 μl of DNA at 75 ng/μl was used for SNP genotyping using the 1536 plex assay kit and GoldenGate Assay Mouse Medium Density Linkage Array following the manufacturer’s protocol (Illumina, San Diego, CA). The intensity data for each SNP for 307 tail DNA samples were normalized and the genotypes assigned using Illumina GenomeStudio Genotyping Analysis Module version 1.9.4. SNPs with a GC score < 0.7 and non-informative (homozygous) SNPs were excluded from further analysis. SNP Hardy–Weinberg equilibrium (HWE) *p*-values were estimated with PLINK. SNPs were omitted if the HWE *p* < 0.001. QTL mapping was performed for all traits using a single-QTL analysis in J/qtl [24]. For binary traits a binary model was used all other traits were analyzed using a non-parametric model. Permutation testing [17] of 10,000 permutations was used to determine significance. Age of death of (TRAMP x WSB) F2 mice was included as an additive covariate for assessment of primary tumor traits (prostate tumor burden and seminal vesicle tumor burden). Age of death and prostate tumor burden were used as additive covariates for assessment of all distant metastasis-related traits (lung, lymph node and liver). Confidence intervals of all QTLs were determined using 2-LOD support intervals on the chromosome where the LOD score did not fall below 2.0 of its maximum [25]. QTLs reaching a genome-wide α < 0.05 were considered for further evaluation.

### Expression QTL and transcript-trait correlations analyses of mouse prostate tumor using RNA-seq

Methods of RNA-seq analysis described here have been performed in previously published work [16, 18]. Details of the eQTL analyses have been described previously [26]. Total RNA extractions from *n* = 92 (TRAMP x WSB) F2 mouse prostate tissue were carried out using the RNeasy mini kit (QIAGEN) according to the manufacturer’s protocol. Prostates that were small (< 1 g) at the time of collection had RNA extracted from the ventral prostate, since this is the region where neuroendocrine tumors originate in TRAMP mice [27]. RNA quantity was measured using the NanoDrop 2000 (Thermo Scientific, Inc., Waltham, MA) and RNA quality was confirmed using the Bioanalyzer (Agilent, Inc., Santa Clara, CA). RNA-seq libraries were constructed from 1 μg total RNA after rRNA depletion using Ribo-Zero GOLD (Illumina). The Illumina TruSeq RNA Sample Prep V2 Kit was used according to the manufacturer’s instructions. The cDNAs were fragmented to ~ 275 bp using a Covaris E210, amplified for 10 cycles, and optimized for input amount to minimize the chance of over-amplification. Unique barcode adapters were applied to each library for cataloging, which were pooled for sequencing. The pooled libraries were sequenced on multiple lanes of a HiSeq 2500 using version 4 chemistry with a minimum of 43 million 126-base read pairs. The RNA-seq output data was processed using RTA version 1.18.64 and CASAVA 1.8.2.

The first seventeen 5′ bases and the 3′-most base of each raw RNA-seq read was trimmed using Trimmomatic [28]. To map RNA-seq reads from (TRAMP x WSB) F2 mice, Seqnature [29] was used to generate a diploid genome sequence that contained C57BL/6 and WSB sequence variation and an allele-specific transcriptome was generated. Bowtie2 was used to map RNA-seq reads to the allele-specific transcriptome, and RSEM was used to quantify the RNA-seq data. eQTL analysis was performed using Matrix-eQTL in R [26]. To test for associations between gene expression and SNPs from QTL mapping, we employed a linear model using age and primary tumor burden as covariates. Proximal eQTLs were defined as a SNP that mapped ≤1 Mb upstream or downstream of the transcription start site of the gene. Benjamini-Hochberg FDR was used to correct for multiple testing and a false discovery rate (FDR) < 0.05 was considered significant. Using MedCalc (Ostend, Belgium) Pearson correlation coefficients and *p*-values were calculated for phenotypic traits in (TRAMP x WSB) F2 mice by correlating log2 transformed expression intensities of all probes mapped to a relevant QTL trait. Student’s t-tests were used to determine the significance of transcript-trait correlations. Benjamini-Hochberg FDR method using the QVALUE module in R was used to correct for multiple testing [30]. Significant correlations were considered at FDR < 0.05.

Candidate genes were nominated by performing the analyses described above for all of the transcripts physically located within the boundaries of a QTL for a given trait. Candidate gene nominated for further analysis had both of the following characteristics: 1) a *cis*-eQTL; and 2) an expression level correlated with the relevant aggressive disease trait.

### Identifying variants associated with clinical outcomes of PC in human GWAS

Any associations of variants with aggressive traits of PC were characterized in two human GWAS cohorts: The Cancer Genetic Markers of Susceptibility (CGEMS) GWAS with 1172 PC cases of varying degrees of aggressiveness [31], and the International Consortium for Prostate Cancer Genetics (ICPCG) GWAS of familial PC with 2568 cases [32]. Association analyses described here have been performed in previously published work [16, 18]. SNPs located within 100 kb of either the transcription start site (TSS) or transcription end site (TES) for each candidate gene identified from the (TRAMP x WSB) F2 mouse studies (Additional file 1; highlighted in bold) were analyzed in both GWAS cohorts, since the highest density of disease-associated cis-eQTL variants fall within a 100 kb radius [33]. Hardy-Weinberg equilibrium *p*-values were estimated using PLINK [34]. SNPs and genes were mapped to GRCh37/hg19 and any SNPs with *p* > 0.01 were excluded from further analyses. For the CGEMS cohort, associations between aggressive PC clinical traits and SNP frequency were defined using the following comparisons: 1) pathological stage I + II versus stage III + IV; 2) tumor stage T1 + T2 versus T3 + T4; 3) nodal metastasis N0 versus N1 + N2; 4) distant metastasis M0 versus M1A + M1B + M1C; and 5) Gleason score < 7 versus > 7. For the ICPCG, cases were pre-coded in dbGAP and variant frequencies were compared between cases coded ‘aggressive’ and cases coded ‘non-aggressive’ (sum of moderate and insignificant disease) as described previously [35].

Associations between aggressive PC traits and SNP were determined using a generalized linear model (GLM). Age, PC1, PC2 and PC3 were incorporated as covariates when performing GLM analysis. Correction for compounding of type I error was performed using a permutation test [17] using the GLM on National Institutes of Health (NIH) Biowulf super cluster computer system (https://hpc.nih.gov/). Permutation testing (*n* = 10,000 permutations) was carried out by rearranging phenotype labels for SNPs in the same linkage disequilibrium (LD) block for each subject, when nominal *p* < 0.010. Genome-wide LD blocks were estimated by using the Solid Spine algorithm of Haploview [36] with standard parameters. All these analyses were performed using R and genes harboring significant associations were used for further downstream analysis.

### Identifying candidate genes with differential mRNA expression in human PC gene expression cohorts

Correlation of candidate gene expression levels with aggressive PC clinical variables was analyzed in three PC gene expression datasets: The Cancer Genome Atlas [TCGA] prostate adenocarcinoma [PRAD], GSE46691 and GSE21032. TCGA PRAD dataset consists of RNA-seq data of *n* = 499 PC cases; GSE46691 and GSE21032 consists of microarray data of *n* = 545 and *n* = 150 PC cases, respectively. Logistic regression analysis was performed using MedCalc (Ostend, Belgium) to identify any associations of gene expression levels of each transcript uncovered from our GWAS analysis, with divergent aggressive PC clinical outcomes. PC clinical traits were sorted into ‘aggressive’ and ‘non-aggressive’ based on the following characteristics: for pathological stage, stage I + II versus stage III + IV; for tumor stage, T1 + T2 versus T3 + T4; for nodal metastasis, N0 versus N1 + N2; for distant metastasis, M0 versus M1A + M1B + M1C; for Gleason score, < 7 versus > 7; and for biochemical recurrence, recurrent versus non-recurrent. Candidate gene expression levels are presented as z-scores. For TCGA; z-scores were generated from RNA-seq read counts by calculating the standard deviation (SD) of transcript expression levels in each case compared to the mean transcript expression in tumors. For GSE46691, z-scores were calculated using microarray gene expression data, by calculating the SD of the levels of transcript in each case compared to the mean transcript expression in all tumors. Finally, z-scores in GSE21032 were calculated by generating SDs for the comparison of mean transcript expression in cases compared to the average transcript expression level in matched normal prostates (*n* = 149). Correction for multiple testing was calculated using Benjamini-Hochberg FDR and for univariate logistic regression *p*-values with the threshold for significance being an FDR of 5%. Kaplan–Meier survival analysis was performed by comparing the survival time in all cohorts with higher or lower levels of tumor candidate gene expression versus all other cases. Higher or lower levels of gene expression were defined by a z-score of > 2 or < − 2, respectively. Significance of survival analyses was performed using the Cox F test. Kaplan–Meier survival analysis was performed by using IBM SPSS Statistic 24 package and plots are presented as cumulative survival over time (months). Next, we determined if each of the candidate genes that exhibited significant correlations with clinical outcomes in TCGA cohort also demonstrated significant differential expression profiles between PC and normal prostate tissue samples in the same cohort. We utilized normalized expression values for each candidate (mouse tissue: RNA-seq TPM counts; TCGA: RNA-seq FPKM counts). The mean ± SD of mRNA expression from 491 PC cases were compared to 52 cases of normal prostate samples and verified using a two-tailed student’s t-test, with significance determined at *p* < 0.05..

### Cell culture and lentiviral-mediated transfection of LNCaP cell lines

We sought to validate our candidates as PC modifier genes using the human prostate adenocarcinoma cell line LNCaP (ATCC CRL-1740), and assessing if ectopic overexpression of each candidate gene could affect parameters of growth, invasion and migration. LNCaP cells were maintained in RPMI media (Gibco) containing 10% fetal bovine serum (FBS) (Sigma) and 1% penicillin/streptomycin antibiotic with mycolplasma testing performed routinely. Lentiviral vectors for *CCDC115* and *DNAJC10* and an equivalent backbone empty control vector were purchased from GE Dharmacon (Lafayette, CO). Lentiviral vectors for *RNF149* and *STYXL1* and an equivalent backbone empty control vector were purchased from Genecopia (Rockville, MD). Lentiviral particles were generated from competent 293 T cells (ATCC, CRL-3216) using Superfect reagent (QIAGEN) according to manufacturer’s instructions. Viral particles were transfected into LNCaP cells for 4 h, with repeated transfection the following day. Two days later, selection for stably transfected cells was performed using 3 mg/mL blasticidin for the empty vector control purchased from Dharmacon (herein referred to as control_B), *CCDC115, DNAJC10,* or 10 mg/ml puromycin for the empty control vector purchased from Genecopia (herein referred to as control_P), *RNF149* and *STYXL1*, for a total of 2 weeks. Successful transfection was confirmed by qPCR and by Western blot using V5 antibody for control_B, CCDC115 and DNAJC10, and HA tag antibody for control_P, RNF149 and STYXL1.

### Growth, invasion, migration and anchorage independent growth assays of LNCaP cell lines over-expressing candidate genes

To measure cell growth we performed whole cell counts daily over 6 days in duplicate. A total of 2.5 × 10^4^ cells per well were seeded in a 12 well plate on day 0. At the same time each day, cells were trypsinized, mixed with full serum media and counted in duplicate using a T4 Cellometer counter (Nexcelom Bioscience LLC, MA). Statistically significant differences in cell growth were determined using ANCOVA (MedCalc) compared to the control. Anchorage independent growth was measured by plating 2 × 10^3^ cells per 24-well in 0.33% bacto-agar in duplicate. Plates were incubated at 37 °C for 14 days where colony forming units were counted in each well. For migration and invasion measurements, cells were starved in serum-free media overnight. A total of 5 × 10^5^ cells were seeded with serum-free media into an 8.0 μM insert membrane (Thermo Scientific, Inc.) retained in a 24 well plate holding 500 μl of media + 10% FBS, which serves as an attractant to the starved cells. Prior to the assay, insert membranes were pre-coated with collagen I for migration assays and with Matrigel (BD Biosciences, San Jose, CA) for invasion assays. Forty-eight hours later, cells that were retained in the upper chamber insert were removed gently with a moist cotton swab. Cells that invaded/migrated to the lower surface were fixed with 4% paraformaldehyde and stained with crystal violet (0.05% in ethanol) with membranes de-stained in 2% SDS. Absorbance was read at 560 nm using a microplate reader (Molecular Devices, Sunnyvale, CA). Statistical analyses of absorbance reading for invasion and migration were performed using Student’s t test (two tailed), and data are presented as mean ± SD where *p* < 0.05 was considered significant.

### Flank xenograft assay in NU/J mice using LNCaP cell lines over-expressing candidate genes

To identify changes in tumor growth, LNCaP cell lines overexpressing our candidate genes of interest or controls (Cohort 1: control_B, *CCDC115, DNAJC10;* Cohort 2: control_P*, RNF149, STYXL1*) were used in flank xenograft experiments. Male nude mice homozygous for Foxn1<nu> aged 6 weeks old were imported from The Jackson laboratories (Bar Harbor, ME, Stock# 002019) and housed in a pathogen free environment with 4–5 mice per cage under controlled conditions of 22 ± 2 °C (SD), 80 ± 10% humidity and 12-h light/dark cycle with daily heath monitoring. Mice were acclimatized for one week and randomized to each treatment group prior to experiments. LNCaP cells were grown with RPMI + 10% FBS in 200 mm plates until they reached approximately 85% confluence. Cells were trypsinized and counted and a total of 2 × 10^6^ cells were re-suspended per 50 μl of PBS on ice. Immediately prior to injection, 50 μl of cell suspension was mixed with 50 μl of ice cold Matrigel® Matrix (Corning, cat#354230) and injected subcutaneously into the flanks of *n* = 8 mice per cell line. Tumor length and width was measured with digital calipers once weekly until tumors reached 200mm^3^ in size, or when mice reached humane endpoints. Tumor volume was calculated by (length2 x width) / 2 and statistical analysis was performed using one-way ANOVA (MedCalc). Tumor weight was recorded in grams and differences between groups were determined using Students two-tailed t-test. Significance was reached at *p* < 0.05. This experiment was replicated once in an additional n = 8 mice with a different passage of LNCaP cells.

## Results

### Modifier locus mapping of (TRAMP x WSB) F2 mice reveal seven genomic loci associated with aggressive disease burden

**Fig. 2:**
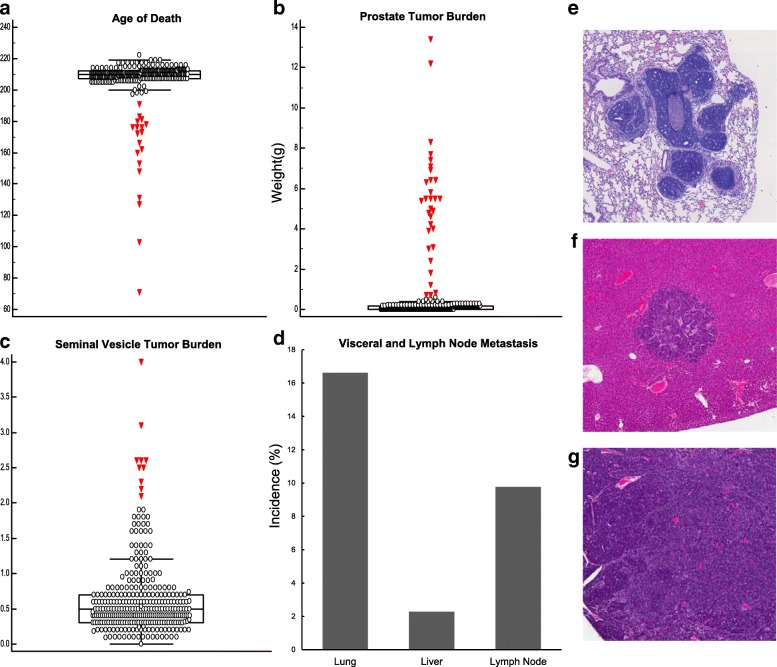
Summary of phenotypic data collected from *n* = 307 (TRAMP x WSB) F2 transgene positive mice. At 210 days, or when human end points were reached, mice were scarified and tissues collected and analyzed for primary tumor burden and distant metastases. **a** Age of death. **b** Prostate tumor burden. **c** Seminal vesicle tumor burden. **d** Visceral and lymph node metastasis (Incidence %)**.** Representative H&E staining. **e** Lung. **f** Liver. **g** Lymph node

**Fig. 3:**
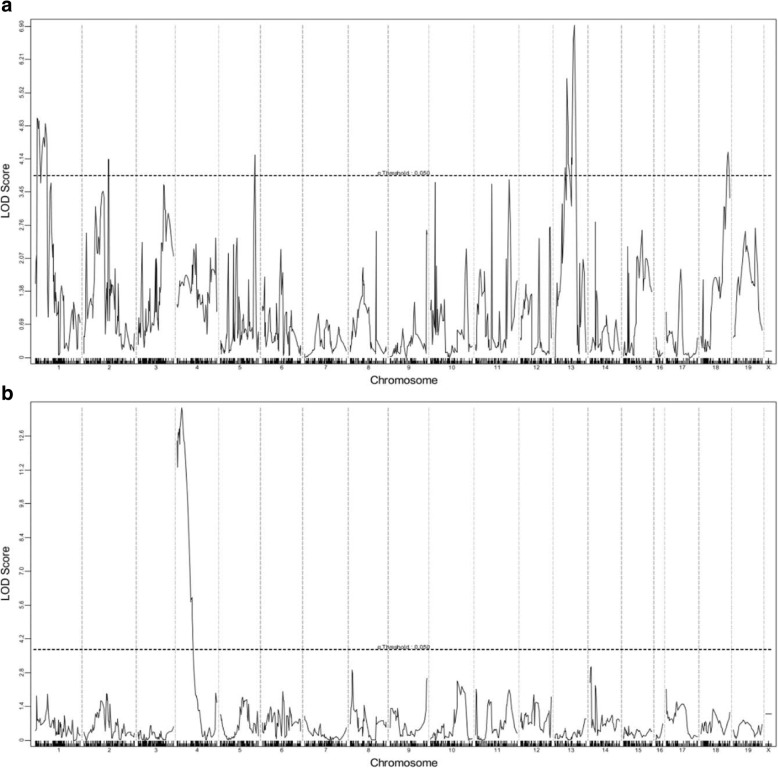
Genome wide QTL plots of significant modifier loci in (TRAMP x WSB) F2 mice. **a** Prostate tumor burden. **b** Seminal vesicle tumor burden

After 210 days, or when humane endpoints were reached, (TRAMP x WSB) F2 mice were euthanized and assessed for primary tumorigenesis and metastasis (Fig. 2). The average age of death was 207 days ±15 days (Fig. 2a), where *n* = 12 mice died prior to the designated endpoint of 210 days, the average prostate tumor burden was 0.59 g ± 1.80 g (Fig. 2b) and average seminal vesicle tumor burden was 0.64 g ± 0.54 g (Fig. 2c). Lung metastases were the most common site of distant metastasis with 51/307 mice (16.6%), followed by lymph node metastases in 30/307 mice (9.77%), and liver metastases in 7/307 mice (2.2%) (Fig. 2d). Metastasis was confirmed by H&E staining (Fig. 2e-g). QTL mapping was performed by correlating these phenotype data with germline SNP data, with 729 informative SNPs in this F2 cross, revealing 7 genomic loci that were associated with aggressive disease traits that reached genome-wide significance of *p* < 0.05 (summarized in Table 1). Loci on chromosomes 1, 2, 5, 13 and 18 were associated with prostate tumor burden, and one locus on chromosome 4 was associated with seminal vesicle tumor burden. QTL plots of each of these traits are presented in Fig. 3. There were no significant QTLs identified for metastasis related traits.

**Table 1:** Significant aggressive PC susceptibility modifier loci in (TRAMP x WSB) F2 mice

Trait	Chromosome	LOD Score	P-Value	2-LOD Confidence Interval	
				Proximal (bp)	Distal (bp)
Primary Tumor Burden	1	4.96	0.004	16,278,642	64,873,174
	2	4.13	0.026	44,988,302	107,465,137
	5	4.22	0.023	129,258,884	139,764,653
	13	6.92	< 0.001	60,718,040	98,858,290
	18	4.28	0.02	73,432,362	88,633,996
Seminal Vesicle Tumor Burden	4	13.77	< 0.001	3,722,677	46,175,356

*bp* base pair

### RNA-seq identifies fourteen genes harboring cis-eQTLs and expression correlations with prostate tumor burden

Prostate samples (92/307) were collected randomly from (TRAMP x WSB) F2 mice at the time of euthanasia and analyzed by RNA-seq. Locally acting expression QTL (*cis*-eQTL) that had an influence on primary tumor burden were determined, and correlations between prostate tumor expression and disease burden also assessed. A total of 25 candidate genes at four specific loci harbored both a significant *cis*-eQTL and a significant mRNA expression correlation with prostate tumor burden (PTB) in (TRAMP x WSB) F2 mice (Additional file 1). There were no significant genes harboring both a significant cis-eQTL and expression correlation for seminal vesicle tumor burden. Of these 25 genes, only fourteen genes (Additional file 1; highlighted in bold) could be identified as having a human ortholog, with eleven other genes not having any human ortholog. Since our primary aim is to determine the influence of hereditary variation on human disease outcomes, the eleven genes that did not have a human ortholog were not considered for further evaluation. Of the fourteen genes remaining genes, seven candidates were located on Chromosome 1 (C*cdc115, Gsta3, Kcnq5, Ogfrl1, Pkhd1, Gm15832, and Slc9a2*), three were on Chromosome 2 (*Dnajc10, Nup35, and Tfpi*), one on Chromosome 5 (*Styxl1*), and three on Chromosome 13 (*Glrx, Zfp87, Zfp738*). All of these candidate genes were analyzed further in human PC cohorts.

### Validation of (TRAMP x WSB) F2 candidate genes using human GWAS datasets identifies four modifier genes of interest

Clinical relevance of the fourteen candidate genes identified in the (TRAMP x WSB) F2 mouse study to human disease was determined using a three-stage in silico validation: 1) Mining GWAS cohorts to find associations of known genomic variants within genes of patients with clinically aggressive disease traits; 2) analyzing large tumor expression datasets to find any association of aberrant gene expression of patients displaying aggressive clinical traits of PC; and 3) Identify those genes harboring aberrant expression levels between non-tumor, normal prostate samples and prostate adenocarcinoma in human datasets.

In the first stage, we analyzed human PC GWAS to determine whether any of the fourteen candidate genes harbored SNPs associated with aggressive PC. To achieve this aim, we utilized two human GWAS cohort: a) CGEMS (*n* = 688 aggressive and 484 non-aggressive cases); and b) ICPCG (*n* = 1398 aggressive and 1117 non-aggressive cases). SNPs were mapped within a 100 kb radius of each candidate genes, and allele frequencies compared between aggressive and non-aggressive cases. Of the fourteen candidate genes, eleven harbored one or more SNP alleles associated with a differential susceptibility to aggressive disease (*CCDC115, DNAJC10, GLRX, GSTA3, KCNQ5, OGFRL1, PKHD1, STYKL1, RNF149, ZNF729, and ZNF502*) in either GWAS cohort. Specifics of the variants, clinical traits, odds ratios, *p* values and permutation values are presented in detail in Table 2.

**Table 2:** Associations between aggressive disease occurrence and SNPs using case-only analyses of two publicly available human prostate cancer GWAS

Cohort	Gene	Chr.	Clinical Trait	SNP ID	t value	P value	OR (95% CI)	Permutation P value^a^
CGEMS	*CCDC115*	2q21.1	Gleason Score	rs11542411	3.05	0.0023	1.21 (1.07–1.36)	0.0024
	*DNAJC10*	2q32.1	Gleason Score	rs288324	−2.74	0.0062	0.87 (0.80–0.96)	0.0049
	*ZNF502*	3p21.31	Metastasis Stage	rs13321717	2.82	0.0049	1.05 (1.02–1.09)	0.005
	*GLRX*	5q14	Gleason Score	rs154447	2.85	0.0045	1.17 (1.05–1.30)	0.005
	*PKHD1*	6p12.2	Gleason Score	rs1266922	2.98	0.0029	1.17 (1.06–1.30)	0.0026
				rs4711987	−2.72	0.0067	0.87 (0.79–0.96)	0.0072
				rs9382070	−2.65	0.0081	0.88 (0.80–0.97)	0.0081
				rs10948675	−2.64	0.0083	0.88 (0.80–0.97)	0.0061
				rs1937147	−2.64	0.0083	0.88 (0.80–0.97)	0.0085
			Metastasis Stage	rs10484879	−2.95	0.0033	0.94(0.90–0.98)	0.0039
			Tumor Stage	rs10484879	−3.07	0.0022	0.93 (0.88–0.97)	0.0023
				rs9370043	−2.77	0.0056	0.60 (0.41–0.86)	0.0048
				rs1567215	−2.64	0.0083	0.61 (0.42–0.88)	0.0071
				rs1326585	−2.60	0.0096	0.62 (0.43–0.89)	0.0104
	*OGFRL1*	6q13	Tumor Stage	rs12200732	−2.90	0.0038	0.89 (0.82–0.96)	0.0025
	*KCNQ5*	6q14	Gleason Score	rs9442812	−3.05	0.0023	0.65 (0.49–0.86)	0.0035
				rs9351947	−2.73	0.0064	0.85 (0.76–0.96)	0.0059
				rs9341399	−2.64	0.0085	0.58 (0.39–0.87)	0.0079
				rs6952753	2.65	0.0081	1.32 (1.08–1.62)	0.0087
			Nodal Stage	rs9442812	3.61	0.0003	1.09 (1.04–1.14)	0.0004
			Tumor Stage	rs10046418	−2.97	0.0030	0.49 (0.31–0.78)	0.0036
	*STYXL1*	7q11.23	Tumor Stage	rs2840794	−2.88	0.0041	0.50 (0.31–0.80)	0.0036
ICPCG	*ZNF729*	19p12	Aggressive vs. non-aggressive	rs283168	2.65	0.0080	1.16 (1.04–1.30)	0.0087
	*RNF149*	2q11.2	Aggressive vs. non-aggressive	rs11677690	−2.64	0.0084	0.87 (0.78–0.96)	0.0096
	*GLRX*	5q14	Aggressive vs. non-aggressive	rs871775	−2.60	0.0093	0.83 (0.72–0.95)	0.0097
	*GSTA3*	6p12.1	Aggressive vs. non-aggressive	rs9474334	−2.59	0.0095	0.88 (0.80–0.97)	0.0086
	*PKHD1*	6p12.2	Aggressive vs. non-aggressive	rs1413917	2.86	0.0043	1.22 (1.06–1.40)	0.0062
	*KCNQ5*	6q14	Aggressive vs. non-aggressive	rs9446848	−3.14	0.0017	0.66 (0.51–0.85)	0.0016
				rs9442891	−3.12	0.0018	0.66 (0.50–0.86)	0.0023
				rs7772526	−3.11	0.0019	0.75 (0.63–0.90)	0.0022
				rs6453613	3.04	0.0024	1.18 (1.06–1.31)	0.0023
				rs6911751	−2.85	0.0044	0.77 (0.64–0.92)	0.0047
				rs9446844	−2.84	0.0045	0.78 (0.65–0.92)	0.0047
				rs6933440	−2.84	0.0046	0.78 (0.65–0.92)	0.0048
				rs7748968	−2.82	0.0048	0.69 (0.53–0.89)	0.0054
				rs9343009	−2.77	0.0056	0.78 (0.66–0.93)	0.0057
				rs1935530	−2.65	0.0081	0.79 (0.66–0.94)	0.007

^a^LD-block wide correction

### Analysis of (TRAMP x WSB) F2 candidate genes in human tumor gene expression datasets

To further investigate the relevance of these eleven genes with aggressive PC development, logistic regression analysis was performed using tumor expression data derived from three datasets: a) TCGA cohort of human PC (*n* = 499); b) GSE21032 (*n* = 149); and c) GSE46691 (*n* = 545). These analyses revealed five of eleven candidate genes *CCDC115, DNAJC10, RNF149, STYXL1, and ZNF502* that harbored significant associations with aggressive disease traits in TCGA cohort only (Table 3 and Additional file 2). No significant associations between candidate gene expression and aggressive disease were observed in either the GSE21032 or GSE46691 tumor gene expression datasets. For the TCGA, expression of two out of five genes (*RNF149* and *ZNF502*) revealed an increase in disease burden in PC patients, their expression was associated with increasing tumor stage for *RNF149* (odds ratio (OR) = 1.42 [1.12–1.79], *p* = 0.0034) and increasing Gleason score for *ZNF502* (OR = 1.42 [1.16–1.74], *p* = 0.0006). In contrast, three candidate genes *CCDC115*, *DNAJC10* and *STYXL1* all demonstrated a decrease in disease burden. *CCDC115* expression was associated with improved disease free survival (OR = 0.67 [0.53–0.85], *p* = 0.0007) and lower Gleason score (OR = 0.67 [0.55–0.81], *p* = 0.0063), *DNAJC10* expression was associated with a lower tumor stage (OR = 0.73 [0.58–0.91], *p* = 0.0001), lower Gleason score (OR = 0.56 [0.44–0.71], p = 0.0001) and improved disease free survival (OR = 0.63 [0.46–0.88], *p* = 0.0066), and *STYXL1* expression was associated with lower Gleason Score (OR = 0.68 [0.55–0.85], p = 0.0007), lower tumor stage (OR = 0.69 [0.55–0.86], *p* = 0.0009) and fewer nodal metastases (OR = 0.57 [0.40–0.80]), *p* = 0.0012).

**Table 3:** Regression correlation of mRNA expression with clinical traits of aggressive PC in the TCGA cohort: Five candidate genes harbor significant expression associations with aggressive disease traits

Gene	Clinical trait	OR (CIs)	P value	FDR
*CCDC115*	Disease Free Survival	0.67 (0.53–0.85)	0.0007	0.020
	Gleason Score	0.73 (0.58–0.91)	0.0063	0.094
*DNAJC10*	Tumor Stage	0.67 (0.55–0.81)	0.0001	0.007
	Gleason Score	0.56 (0.44–0.71)	0.0001	0.007
	Disease Free Survival	0.63 (0.46–0.88)	0.0066	0.094
*RNF149*	Tumor Stage	1.42 (1.12–1.79)	0.0034	0.061
*STYXL1*	Gleason Score	0.68 (0.55–0.85)	0.0007	0.020
	Tumor Stage	0.69 (0.55–0.86)	0.0009	0.021
	Nodal Metastasis	0.57 (0.40–0.80)	0.0012	0.025
*ZNF502*	Gleason Score	1.42 (1.16–1.74	0.0006	0.020

**Fig. 4:**
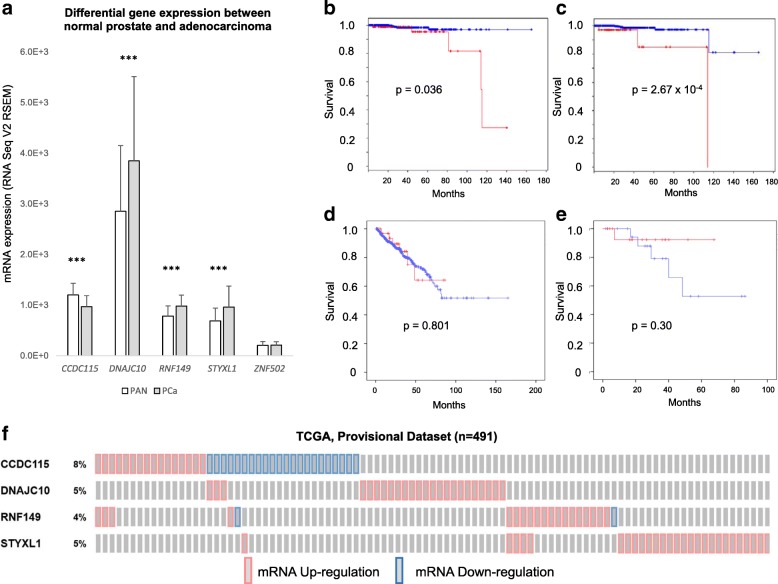
TCGA Cohort of candidate gene expression profiles. **a.** Comparison of differential expression between normal prostate (PAN) Vs adenocarcinoma tissue (PCa) for five candidate genes. **b.** Survival plot for cases with dysregulation of all 4 candidate genes *CCDC115, DNAJC10, RNF149* and *STYXL1* (red) compared to cases with normal expression (blue). **c.** Survival plot of cases with upregulated *DNACJ10* expression (red) compared to cases with normal *DNAJC10* expression levels (blue). **d.** Survival plot of cases with *CCDC115* dysregulation (red) compared to all cases without *CCDC115* differential expression (blue). **e.** Survival plot of those cases with dysregulated CCDC115 expression comparing loss of *CCDC115* expression (blue) and upregulated *CCDC115* expression (red). **f.** Oncoprint showing prostate tumor expression changes in individual cases for each candidate gene (red = upregulated; blue = down regulated). ***p < 0.001

Lastly, out of these five genes that were found to have variants and gene expression changes associated with clinical disease traits, we determined whether their expression was also different between normal and tumor tissue in TCGA cohort. Four out of the five candidate genes *CCDC115, DNAJC10, RNF149* and *STYXL1*, demonstrated significantly differential expression between normal and prostate adenocarcinoma tissue in humans (Fig. 4a). *DNAJC10, RNF149* and *STYXL1* all harbored significantly higher mRNA expression levels in tumor tissues compared to normal prostate. Whereas, *CCDC115* was the only gene to show a loss of mRNA expression in tumor tissue compared to normal. Oncoprints showing aberrant expression of each individual from the TCGA cohort (Fig. 4f) show that *DNAJC10, RNF149* and *STYXL1* were mostly found to be upregulated in PC patients, with the exception of 2 individuals. This is consistent with our finding of higher expression levels found between normal and tumor tissue in Fig. 4a. The most commonly dysregulated candidate gene (8% of all cases) was *CCDC115*, where its expression was up-regulated in approximately 40% of cases but down-regulated in the other 60% of cases (Fig. 4f).

We further determined if dysregulation of any of these 4 candidate genes had any impact on survival probability in TCGA. GSE21032 and GSE46691 were excluded from these analyses since no associations were observed on logistic regression analysis in either cohort. Kaplan-Meier (KM) analysis was applied to compare between cases with aberrant gene expression and those that are normally expressed. KM analyses revealed a significant difference in overall survival between cases with aberrant gene expression (all 4 genes) and cases with normal expression (*p* = 0.036; Fig. 4b). When we examined the effects of each individual gene and their impact on survival probability, we found all patients harboring higher expression levels of *DNAJC10* did not survive, whereas approximately 80% of individuals with normal *DNAJC10* expression were still surviving (*p* = 2.67 × 10^− 4^; Fig. 4c). Although KM analysis of *CCDC115* dysregulation revealed no difference of survival compared to individuals with normal *CCDC115* expression (Fig. 4d), when we separated those individuals according to up- or down-regulated expression levels of *CCDC115,* there was an apparent contrast in survival (Fig. 4e). Patients with higher levels of *CCDC115* survived much longer than those who lost *CCDC115* expression, although this did not reach significance (*p* < 0.30), likely due to a reduced sample size (from *n* = 491 to *n* = 37).

### Functional validations of candidate genes in vitro and in vivo reveal CCDC115 as a biologically important PC modifier gene

**Fig. 5:**
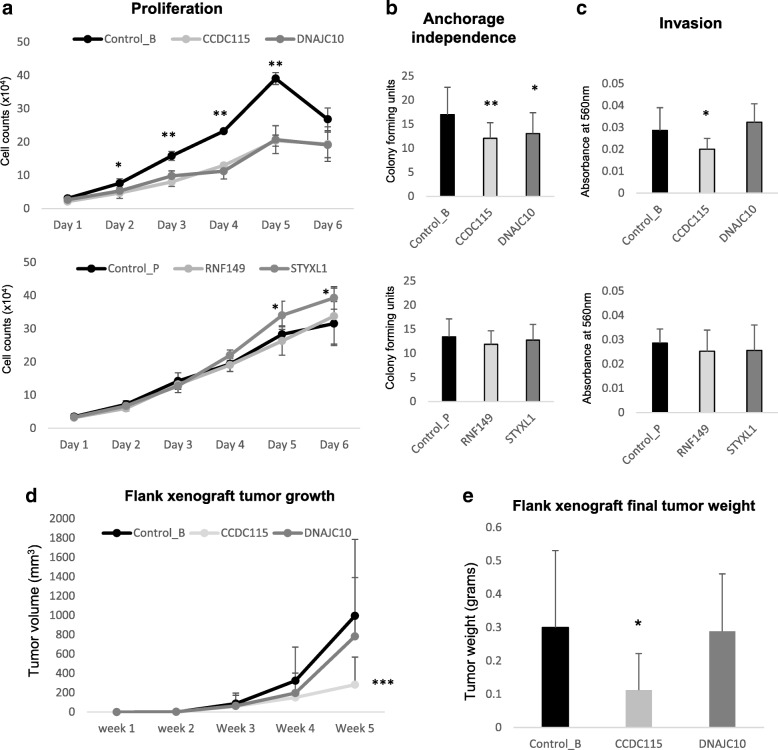
Lentiviral ectopic over-expression of candidate genes *CCDC115, DNAJC10, RNF149* and *STYXL1* in the LNCaP PC cell line and their functional effect in vitro and in vivo*.* **a** Cell proliferation rates. **b** Anchorage independent growth. **c** Invasion. **d** Flank xenograft tumor growth over time. **e** Final tumor weight after 5 weeks growth. *p < 0.05; ** *p* < 0.01; ***p < 0.001

The PC cell line LNCaP was transfected with each one of the five candidate genes of interest, or an empty control vector, using lentiviral mediated transfection. Validation of ectopic overexpression was confirmed by q-PCR (Additional file 3A) and by Western Blot using primary antibodies against the plasmid marker V5 (Control_B, CCDC115 and DNAJC10) or HA tag (Control_P, RNF149 and STYXL1) (Additional file 3B). Cell proliferation counts (Fig. 5a) revealed that after 2 days *CCDC115* and *DNAJC10* can significantly impeded growth of LNCaP cells compared to the control. Whereas *STYXL1* showed increased cell growth after 5 and 6 days and *RNF149* showed no significant difference to control (Fig. 5a). Both *CCDC115* and *DNAJC10* significantly impeded anchorage independent growth after 2 weeks of incubation compared to the control (Fig. 5b). However, no difference in growth was observed for *RNF149* or *STYXL1* (Fig. 5b). *CCDC115* overexpression could significantly impair the invasive potential of LNCaP cells compared to control (Fig. 5c). However, all other genes *DNAJC10*, *RNF149* and *DNAJC10* did not show any significant difference in invasive potential compared to control cells (Fig. 5c). There was no significant difference in cell migration across the membrane for any of the candidate genes compared to the control cells (Additional file 3C).

In order to test the ability of each candidate gene to have an effect on LNCaP cell growth in a more relevant physiological environment, flank xenograft assays were performed in immunocompromised mice. After 5 weeks of measuring tumor growth in 8/8 mice, *CCDC115* overexpression could significantly reduce tumor volume (Fig. 5d) and tumor weight (Fig. 5e) compared to the control group. These findings were replicated in a second cohort of mice (*n* = 8) and the same observation was found (Additional file 4A). None of the other genes *DNAJC10, RNF149* or *STYXL1* had a significant effect on tumor growth or tumor weight (Additional file 4B).

## Discussion

Modifier locus mapping in (TRAMP x WSB) F2 mice has uncovered genomic loci that are associated with primary tumor burden. An in silico validation pipeline using systems genetics approaches, comprising large GWAS and human tumor gene expression datasets, defined modifier genes residing within these loci which influence aggressive disease in a clinically relevant context. These analyses led us to functionally characterize the effects of ectopic expression of the *CCDC115* gene, which we demonstrate as actively inhibiting tumor growth and invasion of a human adenocarcinoma cells in vitro. This is in agreement with our in silico analyses showing *CCDC115* as a PC suppressor. Specifically, this gene harbored significant regression correlation of mRNA expression with clinical traits of aggressive PC, including Gleason score and disease free survival.

This study endeavored to identify nominally associated variants that would not be evident as modifiers of aggressive PC in a traditional GWAS analysis. The GWAS analysis performed here represents another line of evidence implicating the identified candidate genes as being associated with aggressive PC. Uncovering germline susceptibility genes is challenging using already established approaches such as GWAS, with most complex diseases exhibiting varying degrees of “missing heritability”. Indeed, our study here is a clear example of how a gene that has significant biological effects on PC aggressiveness is not flagged as significant genome wide. The systems genetic approach we employ here, and in other studies [15, 16, 18], provides a sophisticated and concise example of how encompassing high resolution modifier locus mapping in mice can uncover novel modifier genes of aggressive PC, where germline variation is known to influence disease aggressiveness [37, 38]. Such approaches will enable discovery of novel susceptibility genes linked to differential outcomes in complex diseases that would otherwise be missed. There are logical reasons for why these genes might be overlooked in traditional approaches of assessing the effects of complex genetic inheritance, including overestimating the scale of complex disease heritability, underestimating allelic effect sizes, and exceptionally rare variants that encompass large effect sizes [18].

A limitation of the F2 cross we employ in the current study are the large mapping intervals encompassing several hundred genes across one-quarter to one-half of a chromosome [39], particularly compared to complex trait mapping in highly genetically diverse mice [18]. Although integrating eQTL and trait correlation analyses using prostate RNA-seq data narrowed the list dramatically to 25 candidate genes, biologically important gene discovery is restricted since transcriptomic analysis was only performed in 92 of the 307 (TRAMP x WSB/EiJ) F2 prostates. Additionally, this candidate gene identification strategy focused on the discovery of modifiers that act through expression-related mechanisms, and therefore does not exclude other types of variants that might also influence disease outcome. Despite this, modifier locus mapping performed in transgenic mouse models, where confounding variables of human studies such as environmental variation can be controlled for, provide the ideal platform to further unearth biologically relevant germline variants of aggressive disease.

This is the first study to identify *CCDC115* as a modifier gene of aggressive PC. A protein harboring a coiled-coiled domain, CCDC115 is poorly studied with few known functions. A homozygous missense mutation c.92 T > C (p.Leu31Ser) in *CCDC115* leads to abnormal protein glycosylation in the Golgi complex [40], a cell apparatus where proteins are packaged ready for export. Furthermore, *CCDC115* is important for lysosomal degradation of known lysosomal substrates such as epidermal growth factor receptor (EGFR), and is important for stabilizing hypoxia inducible factor 1α (HIF1α) [41], both of which have been implicated as crucial in PC development [42, 43]. In particular, EGF signaling via the EGFR can stimulate tumor cell proliferation and promote bone metastasis in PC [44]. There is also evidence to suggest *CCDC115* can disrupt cell proliferation and apoptosis in neuroblastoma cells of the brain via the FGF2 and MAPK pathway [45], where FGF2 has previously been shown to increase migration, invasion and epithelial to mesenchymal transition in PC cell lines [30]. Collectively, these data indicate that *CCDC115* expression could be impeding tumor growth in our current study via several mechanisms, including disruption of the EGFR or FGF2/MAPK pathways. The current study tested the effect of *CCDC115* overexpression in one human prostate cancer cell line LNCaP, which is of adenocarcinoma origin and is not a neuroendocrine cell line. Since all the human datasets (GWAS and tumour gene expression) in the candidate gene validation pipeline were of adenocarcinoma origin, LNCaP cells were the ideal choice to validate the human data. However, future mechanistic studies to understand the role of *CCDC115* in these PC network pathways are needed in other prostate cancer models, including neuroendocrine disease, to understand its potential role in aggressive PC suppression. Furthermore, additional functional studies in the (TRAMP x WSB) F2 cross would define and corroborate a causal role of *ccdc115* in the suppression of neuroendocrine disease in the TRAMP model.

## Conclusion

We present a novel germline variant in the *CCDC115* gene that has the potential to predict clinical outcome in some human PC patients. Determining men at high risk of fatal PC, a disease that kills over 26,000 men in the USA annually, will be essential to optimizing treatment for those patients likely to have disease progression. It might also identify patients with low-risk disease where minimizing treatment would be ideal, leading to a reduction in morbidity associated with over-treatment. The data presented here provide more evidence to suggest that germline hallmarks of individuals with PC should be taken into account when considering prognosis and therapeutic treatment strategies.

## Additional files

Additional file 1: Gene expression correlation analyses cis-eQTLs associated with (TRAMP x WSB) F2 mice prostate tumor burden genomic QTLs: Fourteen genes harbor significant cis-eQTL and expression correlation with prostate tumor burden. (DOCX 20 kb)

Additional file 2: Logistic regression analysis of eleven candidate genes using tumor expression data derived from TCGA, GSE21032 and GSE46691. Five of eleven candidate genes CCDC115, DNAJC10, RNF149, STYXL1, and ZNF502 that harbored significant associations with aggressive disease traits in TCGA cohort only. (XLSX 17 kb)

Additional file 3: **a** Confirmation of mRNA ectopic overexpression by RT-PCR. **b** Confirmation of protein ectopic overexpression using V5 (top) and HA (lower) tag primary antibodies (Lanes: 1. Control_B, 2. *CCDC115*, 3. *DNAJC10*, 4. Control_P, 5. *RNF149*, 6. *STYXL1*). c Migration. (PDF 357 kb)

Additional file 4: **a** Validation of in vivo flank xenograft experiments using lentiviral ectopic over-expression of candidate genes *CCDC115, DNAJC10, RNF149* and *STYXL1* in the LNCaP PC cell line. **b** Flank xenograft tumor growth over time using lentiviral ectopic over-expression of candidate genes *RNF149* and *STYXL1* in the LNCaP PC cell line. c Flank xenograft final tumor weight after 5 weeks using lentiviral ectopic over-expression of candidate genes *RNF149* and *STYXL1* in the LNCaP PC cell line. (PDF 86 kb)

## Abbreviations

bp: Base pair
CGEMS: The Cancer Genetic Markers of Susceptibility
*cis*-eQTL: Locally acting expression QTL
eQTL: Expression quantitative trait loci
FBS: Fetal bovine serum
FDR: False discovery rate
GLM: Generalized linear model
GWAS: Genome-wide association studies
HWE: Hardy–Weinberg equilibrium
ICPCG: International Consortium for Prostate Cancer Genetics
KM: Kaplan-Meier
LD: Linkage disequilibrium
NEPC: Neuroendocrine prostate cancer
NIH: National Institutes of Health
OR: Odds ratio
PC: Prostate cancer
PSA: Prostate specific antigen
PTB: Prostate tumor burden
QTL: Quantitative trait loci
SD: Standard deviation
SNP: Single nucleotide polymorphism
TCGA: The Cancer Genome Atlas
TES: Transcription end site
Tg: T Antigen
TRAMP: C57BL/6-Tg 8247Ng/J
TSS: Transcription start site
WSB: WSB/EiJ
