# Supplementary material for: Modifier locus mapping of a transgenic F2 mouse population identifies CCDC115 as a novel aggressive prostate cancer modifier gene in humans
Source: BMC Genomics. 2018 Jun 11;19:450. doi: 10.1186/s12864-018-4827-2 (PMC5996485; doi:10.1186/s12864-018-4827-2)
Supplement: Supplementary file 1 — Gene expression correlation analyses cis-eQTLs associated with (TRAMP x WSB) F2 mice prostate tumor burden genomic QTLs: Fourteen genes harbor significant cis-eQTL and expression correlation with prostate tumor burden. (DOCX 20 kb) [file 12864_2018_4827_MOESM1_ESM.docx]

Additional File 1: Gene expression correlation analyses cis-eQTLs associated with (TRAMP x WSB) F2 mice prostate tumor burden genomic QTLs**:** Fourteen genes harbor significant cis-eQTL *and* expression correlation with prostate tumor burden

| **Chr** | **Gene** | **QTL** | **Transcript** | **cis-eQTL** | | | | | | **Correlation** | | | **Human Ortholog** | |
| --- | --- | --- | --- | --- | --- | --- | --- | --- | --- | --- | --- | --- | --- | --- |
|  |  |  |  | **SNP** | **s1** | **Beta** | **t-stat** | **p-value** | **FDR** | **r-value** | **p-value** | **FDR** | **Symbol** | **HGID** |
| **1** | ***Ccdc115*** | **PTB** | **ENSMUSG00000042111** | **rs3681732** | **34,436,670** | **1.30545991** | **3.56632218** | **0.000557** | **0.038027** | **0.27417** | **0.004635** | **0.015993** | ***CCDC115*** | **84317** |
| **1** | ***Gsta3*** | **PTB** | **ENSMUSG00000025934** | **rs6404446** | **21,240,589** | **-10.068138** | **-4.7742562** | **6.18E-06** | **0.001416** | **-0.37364** | **8.54E-05** | **0.00038** | ***GSTA3*** | **2940** |
| **1** | ***Kcnq5*** | **PTB** | **ENSMUSG00000028033** | **rs6404446** | **21,398,403** | **0.43150218** | **4.26617804** | **4.52E-05** | **0.006123** | **0.436869** | **3.1E-06** | **1.62E-05** | ***KCNQ5*** | **56479** |
| **1** | ***Ogfrl1*** | **PTB** | **ENSMUSG00000026158** | **rs6173215** | **23,366,424** | **-1.4249353** | **-3.3228058** | **0.001246** | **0.066931** | **-0.3874** | **4.39E-05** | **0.000202** | ***OGFRL1*** | **79627** |
| **1** | ***Pkhd1*** | **PTB** | **ENSMUSG00000043760** | **rs6404446** | **20,057,779** | **-0.5915289** | **-4.2824968** | **4.25E-05** | **0.005849** | **-0.3689** | **0.000107** | **0.000469** | ***PHKD1*** | **5314** |
| **1** | ***Gm15832*** | **PTB** | **ENSMUSG00000085894** | **rs13475823** | **39,547,570** | **-2.5175305** | **-4.938012** | **3.17E-06** | **0.000871** | **-0.42832** | **5.06E-06** | **2.6E-05** | ***RNF149*** | **284996** |
| **1** | ***Slc9a2*** | **PTB** | **ENSMUSG00000026062** | **rs13475823** | **40,680,574** | **-5.1196505** | **-3.2635805** | **0.001506** | **0.07629** | **-0.58085** | **7.66E-11** | **7.24E-10** | ***SLC9A2*** | **6549** |
| **2** | ***Dnajc10*** | **PTB** | **ENSMUSG00000027006** | **rs3722345** | **80,315,466** | **-101.0983** | **-3.3431298** | **0.001167** | **0.063643** | **-0.58252** | **6.57E-11** | **6.22E-10** | ***DNAJC10*** | **54431** |
| **2** | ***Nup35*** | **PTB** | **ENSMUSG00000026999** | **rs3722345** | **80,617,236** | **4.31420735** | **8.73541753** | **5.80E-14** | **1.03E-10** | **0.597202** | **1.62E-11** | **1.67E-10** | ***NUP35*** | **129401** |
| **2** | ***Tfpi*** | **PTB** | **ENSMUSG00000027082** | **rs8273639** | **84,432,855** | **-6.5831075** | **-5.1713111** | **1.20E-06** | **0.000412** | **-0.5981** | **1.48E-11** | **1.54E-10** | ***TFPI*** | **7035** |
| **3** | ***Styxl1*** | **PTB** | **ENSMUSG00000019178** | **rs13478546** | **135,747,220** | **-0.0383275** | **-3.7359392** | **0.000311** | **0.024876** | **-0.35214** | **0.000228** | **0.000963** | ***STYXL1*** | **51657** |
| **13** | ***Glrx*** | **PTB** | **ENSMUSG00000021591** | **rs13481883** | **75,839,868** | **2.39606931** | **5.75322854** | **9.60E-08** | **4.57E-05** | **-0.32929** | **0.000599** | **0.002393** | ***GLRX*** | **37566** |
| **13** | ***Zfp87*** | **PTB** | **ENSMUSG00000097333** | **rs4229817** | **67,515,781** | **1.97249125** | **4.90056995** | **3.69E-06** | **0.000976** | **-0.2334** | **0.016534** | **0.05045** | ***ZNF502*** | **91392** |
| **13** | ***Zfp738*** | **PTB** | **ENSMUSG00000048280** | **rs4229817** | **67,667,437** | **-2.2924739** | **-13.011533** | **3.07E-23** | **2.54E-19** | **0.325821** | **0.00069** | **0.002743** | ***ZNF729*** | **100287226** |
| 1 | *Gm36949* | PTB | ENSMUSG00000104138 | rs6404446 | 21,454,691 | 0.05169417 | 3.47864268 | 0.000748 | 0.046018 | 0.753684 | 1.32E-20 | 1.01E-18 | none | none |
| 2 | *BB218582* | PTB | ENSMUSG00000085218 | rs13476689 | 106,642,914 | -0.5769984 | -4.1653275 | 6.61E-05 | 0.008021 | -0.57013 | 2.03E-10 | 1.78E-09 | none | none |
| 2 | *Gm13688* | PTB | ENSMUSG00000083284 | rs3722345 | 80,602,733 | 0.09301444 | 5.27810403 | 7.61E-07 | 0.000288 | 0.63092 | 4.86E-13 | 6.48E-12 | none | none |
| 2 | *Gm9821* | PTB | ENSMUSG00000095332 | rs260568942 | 91,945,703 | 0.79411549 | 3.59448841 | 0.000507 | 0.035544 | 0.729791 | 8.26E-19 | 3.84E-17 | none | none |
| 5 | *4933404O12Rik* | PTB | ENSMUSG00000097908 | rs13478546 | 136,919,146 | 1.25895434 | 4.85021786 | 4.54E-06 | 0.001124 | 0.621853 | 1.3E-12 | 1.61E-11 | none | none |
| 5 | *Gm5050* | PTB | ENSMUSG00000106243 | rs13478546 | 136,211,012 | 0.20159919 | 5.1413611 | 1.36E-06 | 0.000451 | 0.529516 | 6.01E-09 | 4.32E-08 | none | none |
| 13 | *9430065F17Rik* | PTB | ENSMUSG00000097360 | rs4229817 | 67,553,234 | -0.2046609 | -8.3859647 | 3.34E-13 | 5.16E-10 | -0.21668 | 0.026363 | 0.074866 | none | none |
| 13 | *Gm26587* | PTB | ENSMUSG00000097470 | rs4229817 | 67,660,457 | -0.5345363 | -11.471474 | 6.08E-20 | 2.51E-16 | 0.310549 | 0.001256 | 0.004806 | none | none |
| 13 | *Zfp429* | PTB | ENSMUSG00000078994 | rs4229817 | 67,389,309 | -0.4664506 | -8.5506799 | 1.46E-13 | 2.42E-10 | -0.38133 | 5.91E-05 | 0.000267 | none | none |
| 13 | *Zfp708* | PTB | ENSMUSG00000100235 | rs4229817 | 67,069,399 | -0.466059 | -4.1355352 | 7.38E-05 | 0.008748 | -0.58333 | 6.08E-11 | 5.85E-10 | none | none |
| 13 | *Zfp874a* | PTB | ENSMUSG00000069206 | rs4229817 | 67,424,549 | -1.0984441 | -7.1119121 | 1.74E-10 | 1.66E-07 | -0.45873 | 8.36E-07 | 4.67E-06 | none | none |

Chr: chromosome; s1: start position; eQTL: expression QTL; FDR: false discovery rate; HGID: Hugo gene nomenclature ID number. Genes harboring a human ortholog are highlighted in bold.
