# Supplementary figures and images for: Modifier locus mapping of a transgenic F2 mouse population identifies CCDC115 as a novel aggressive prostate cancer modifier gene in humans
Source: BMC Genomics. 2018 Jun 11;19:450. doi: 10.1186/s12864-018-4827-2 (PMC5996485; doi:10.1186/s12864-018-4827-2)

## A mRNA over-expression

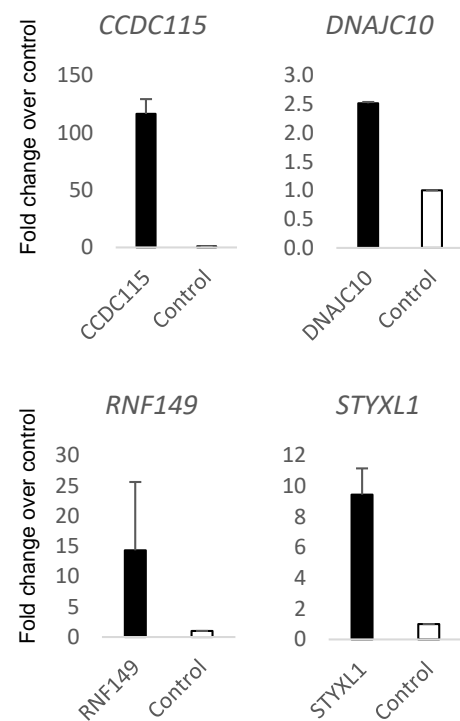

## B Protein over-expression

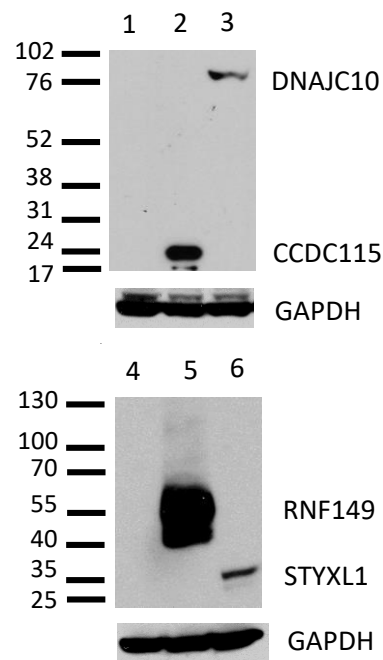

## C Migration

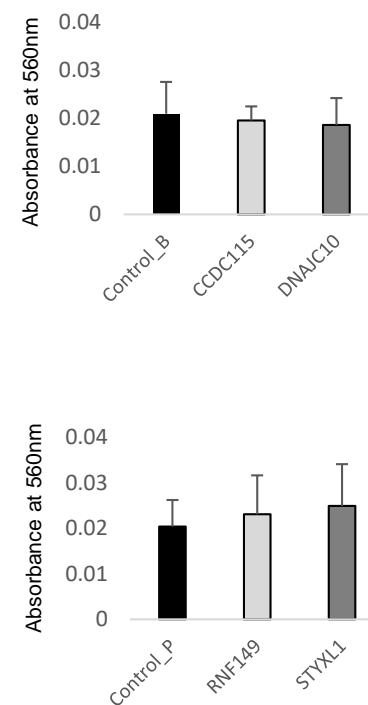

Supplement: Supplementary file 3 — a Confirmation of mRNA ectopic overexpression by RT-PCR. b Confirmation of protein ectopic overexpression using V5 (top) and HA (lower) tag primary antibodies (Lanes: 1. Control_B, 2. CCDC115, 3. DNAJC10, 4. Control_P, 5. RNF149, 6. STYXL1). c Migration. (PDF 357 kb) [file 12864_2018_4827_MOESM3_ESM.pdf]
