# Supplementary material for: Modifier locus mapping of a transgenic F2 mouse population identifies CCDC115 as a novel aggressive prostate cancer modifier gene in humans
Source: BMC Genomics. 2018 Jun 11;19:450. doi: 10.1186/s12864-018-4827-2 (PMC5996485; doi:10.1186/s12864-018-4827-2)
Supplement: Supplementary file 4 — a Validation of in vivo flank xenograft experiments using lentiviral ectopic over-expression of candidate genes CCDC115, DNAJC10, RNF149 and STYXL1 in the LNCaP PC cell line. b Flank xenograft tumor growth over time using lentiviral ectopic over-expression of candidate genes RNF149 and STYXL1 in the LNCaP PC cell line. c Flank xenograft final tumor weight after 5 weeks using lentiviral ectopic over-expression of candidate genes RNF149 and STYXL1 in the LNCaP PC cell line. (PDF 86 kb) [file 12864_2018_4827_MOESM4_ESM.pdf]

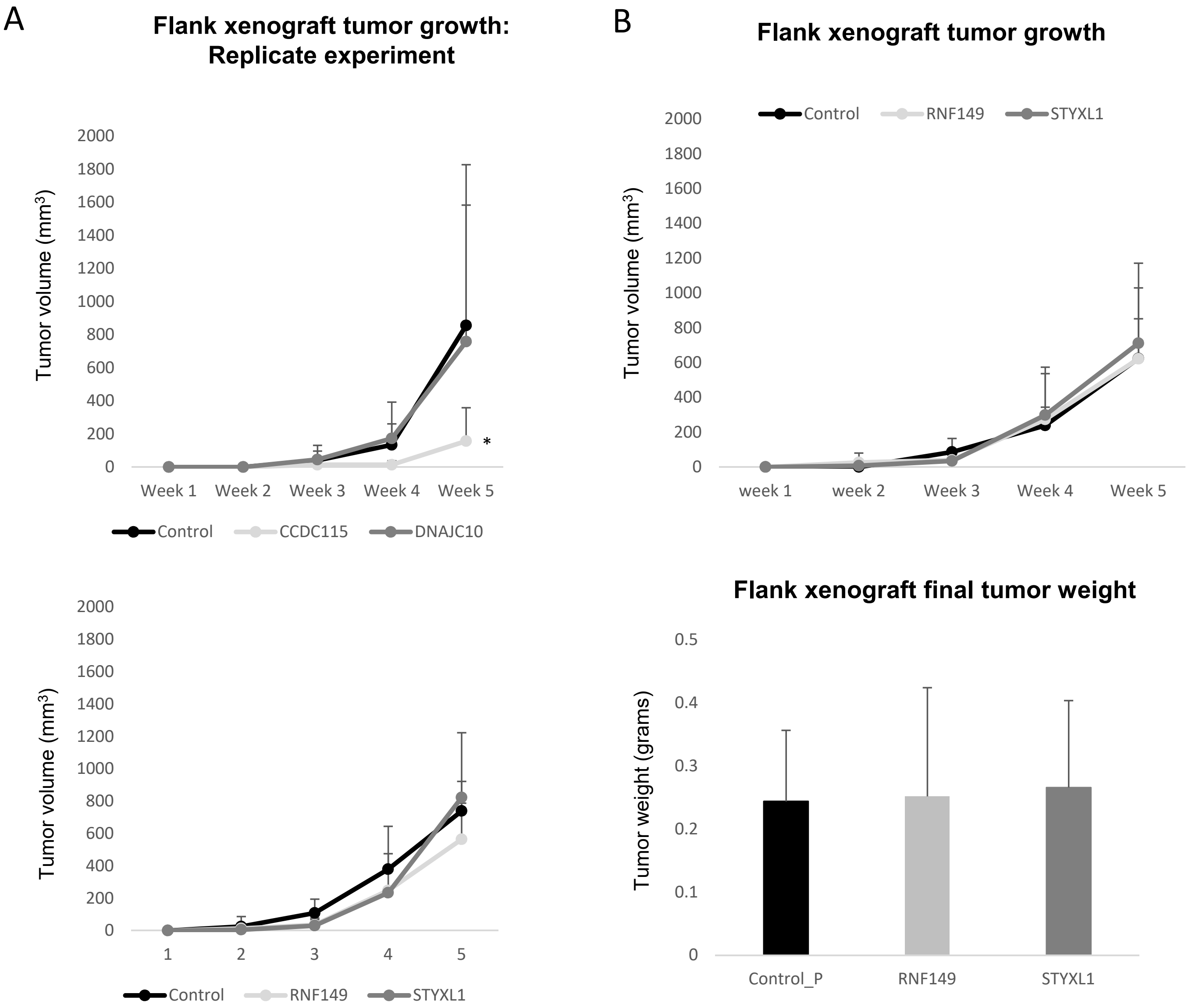
A
Flank xenograft tumor growth: Replicate experiment
Tumor volume (mm3)
2000
1800
1600
1400
1200
1000
800
600
400
200
0
Week 1
Week 2
Week 3
Week 4
Week 5
*
Control
CCDC115
DNAJC10
Tumor volume (mm3)
2000
1800
1600
1400
1200
1000
800
600
400
200
0
1
2
3
4
5
Control
RNF149
STYXL1
B
Flank xenograft tumor growth
Control
RNF149
STYXL1
Tumor volume (mm3)
2000
1800
1600
1400
1200
1000
800
600
400
200
0
week 1
week 2
Week 3
Week 4
Week 5
Flank xenograft final tumor weight
Tumor weight (grams)
0.5
0.4
0.3
0.2
0.1
0
Control_P
RNF149
STYXL1
